# Reproductive Health Surveillance in the US-Mexico Border Region, 2003-2006: The Brownsville-Matamoros Sister City Project for Women’s Health

**Published:** 2008-09-15

**Authors:** Jill A McDonald, Christopher H Johnson, Ruben Smith, Suzanne G Folger, Ana L Chavez, Ninad Mishra, Antonio Hernández Jiménez, Linda R MacDonald, Jorge Sebastián Hernández Rodríguez, Susie Ann Villalobos

**Affiliations:** Centers for Disease Control and Prevention, National Center for Chronic Disease Prevention and Health Promotion, Division of Reproductive Health; Centers for Disease Control and Prevention (CDC), National Center for HIV/AIDS, Viral Hepatitis, STD, and TB Prevention; CDC, National Center for Chronic Disease Prevention and Health Promotion (NCCDPHP), Division of Reproductive Health, and Science Applications International Corporation, Atlanta, Georgia; CDC, NCCDPHP, Division of Reproductive Health, Atlanta, Georgia; CDC, National Center for Health Statistics, Division of Health and Nutrition Examination Surveys, Hyattsville, Maryland; CDC, Office of Workforce and Career Development, Public Health Informatics Fellowship Program, and currently at the National Center of Public Health Informatics, Division of Knowledge Management, Atlanta, Georgia; Instituto Mexicano del Seguro Social, Coordinación Delegacional de Salud Pública, Ciudad Victoria, Tamaulipas, and currently at Coordinación Delegacional de Salud Pública, Aguascalientes, Aguascalientes; University of Texas-Brownsville/Texas Southmost College, Brownsville, Texas; Secretaría de Salud de Tamaulipas, Dirección de Planeación y Coordinación Sectorial, and Comisión de Salud Fronteriza México-Estados Unidos, Sección de México, Ciudad Victoria, Tamaulipas; United States-Mexico Border Health Association, El Paso, Texas

## Abstract

**Introduction:**

High birth and immigration rates in the US-Mexico border region have led to large population increases in recent decades. Two national, 10 state, and more than 100 local government entities deliver reproductive health services to the region's 14 million residents. Limited standardized information about health risks in this population hampers capacity to address local needs and assess effectiveness of public health programs.

**Methods:**

We worked with binational partners to develop a system for reproductive health surveillance in the sister communities of Matamoros, Tamaulipas, Mexico, and Cameron County, Texas, as a model for a broader regional approach. We used a stratified, systematic cluster-sampling design to sample women giving birth in hospitals in each community during an 81-day period (August 21-November 9) in 2005. We conducted in-hospital computer-assisted personal interviews that addressed prenatal, behavioral, and lifestyle factors. We evaluated survey response rates, data quality, and other attributes of effective surveillance systems. We estimated population coverage using vital records data.

**Results:**

Among the 999 women sampled, 947 (95%) completed interviews, and the item nonresponse rate was low. The study sample included 92.7% of live births in Matamoros and 98.3% in Cameron County. Differences between percentage distributions of birth certificate characteristics in the study and target populations did not exceed 2.0. Study population coverage among hospitals ranged from 92.9% to 100.0%, averaging 97.3% in Matamoros and 97.4% in Cameron County.

**Conclusion:**

Results indicate that hospital-based sampling and postpartum interviewing constitute an effective approach to reproductive health surveillance. Such a system can yield valuable information for public health programs serving the growing US-Mexico border population.

## Introduction

**Figure 1.**
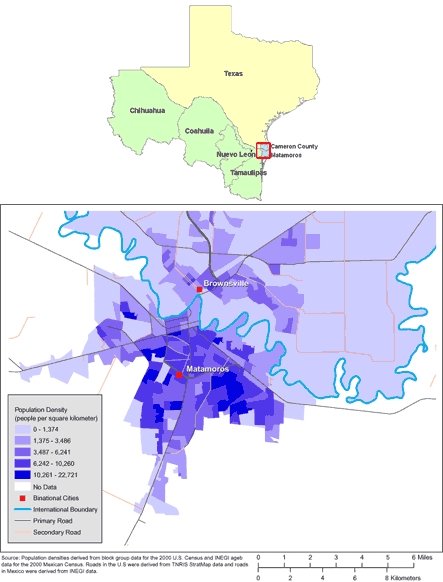
Maps of the US-Mexico Border Region (Top) and of Brownsville, Texas, and Matamoros, Tamaulipas, Mexico (Bottom). (The authors thank Allison Abell Banicki of the Office of Border Health, Texas Department of State Health Services, for creating the map of the Texas-Mexico border states and thank Jean W. Parcher, Sylvia N. Wilson, and the United States Geological Survey [USGS] for providing the map of population density in Brownsville and Matamoros.)

The US-Mexico border region reaches 100 km north and 100 km south of the international divide and is home to 14 million people (1) (Figure 1). Ninety percent of the population resides in 14 pairs of economically and socially interdependent sister cities that lie on the 2,000-mile border (2,3). In 2000, nearly 300,000 births occurred in these paired communities (4). High birth and immigration rates have caused a surge in population in this area in recent decades, and growth is projected to continue through at least 2030 (1).

Information about reproductive health in the border population is scant. Rates of health insurance coverage in US border counties are considerably lower than they are in any US state (5,6), and the shortage of health care professionals is severe (7). Women from US border counties are less likely to receive prenatal care than are women in other counties in US border states, although their risk of infant death and preterm birth appear to be no greater (8,9). Late or no prenatal care is particularly characteristic of adolescents in the region, who have birth rates among the highest in the United States (8,9). In Mexican border communities, adolescent birth rates are also believed to be high, and reducing maternal and infant mortality remain priorities (10,11). Growing concern about sexually transmitted infections and HIV risk is evident on both sides of the border (12-14). US and Mexican border communities share common maternal and child health (MCH) goals for 2010 (15), yet reliable baseline data are not available for many goals and related risk factors. This information is essential for program planning and evaluation.

Multiple factors contribute to the lack of reproductive health data in this dynamic region, including different data collection systems; inconsistent definitions for indicators; uneven distribution of services, such as telephone and mail delivery; low education levels; limited community resources; language barriers; and a mobile population (16-18). To further complicate matters, the region includes 2 national, 10 state, and more than 100 local and regional government entities. On both sides of the border, these factors are obstacles to traditional survey and surveillance methods, which rely on standard definitions for health measures, complete telephone coverage, fixed residences, minimum reading levels, and data sharing among government institutions.

We developed methods for reproductive health surveillance characterized by shared reproductive health goals, strong local and binational partnerships, and a bilingual approach to data collection. Effective data collection methods developed in 1 pair of sister communities can be duplicated in other communities or used as a model for a region-wide approach. We describe the methods and operational results from the pilot test conducted in 1 pair of sister communities in the US-Mexico border region.

## Methods

### Site selection and protocol development

We chose Cameron County, Texas (with the cities of Brownsville and Harlingen), and Matamoros, Tamaulipas, Mexico, as the paired site for this demonstration project because their population size was average among the sister communities (379,000 for Cameron County and 462,000 for Matamoros, in 2005) (19,20) and because of local interest in the project. Starting in 2003, we worked with University of Texas partners in Brownsville to expand partnerships and build support among local health authorities and providers of MCH services. We met with program directors at state health institutions in Texas and Tamaulipas and with the US and Mexican sections of the United States-Mexico Border Health Commission (USMBHC) to encourage their support.

Review of Texas birth records and discussions with health officials in Matamoros showed that most births in the Texas-Tamaulipas border region were occurring in hospitals, indicating that hospital-based sampling and postpartum interviews conducted in hospitals would yield data representative of mothers and infants in these communities. We collected information on patient admissions and labor and delivery record-keeping procedures from each community hospital and used this information to design a procedure to sample and interview women who gave birth to live infants in these communities. We sought input from institutional partners throughout the process and worked closely with the Secretariat of Health in Tamaulipas to develop methods that would later be used to assess population coverage. We met annually from 2003 through 2005 with community stakeholders to discuss progress with protocol development, solicit feedback, and plan next steps. The Brownsville-Matamoros Sister City Project for Women's Health (BMSCP) pilot project was reviewed for human subject concerns by the Centers for Disease Control and Prevention (CDC) and was determined to be "nonresearch" or public health practice. Therefore, institutional review board approval was not required. Training materials and evaluation procedures were completed in July 2005. BMSCP collaborators include government, nongovernment, and academic institutions at the federal, state, and local levels (Table 1).

### Sample design

**Figure 2.**
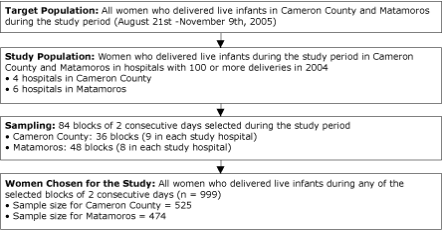
Sampling Design of the Surveillance System Used for the Brownsville-Matamoros Sister City Project for Women's Health, August 21-November 9, 2005.

We used a stratified, systematic cluster-sampling design (Figure 2). The target population was women who gave birth to live infants in Matamoros and Cameron County, and the study population was women who gave birth to live infants in hospitals with 100 or more deliveries in 2004 in each community. A sample size of 500 was planned for each community. Because of the expected numbers of births during the study period and an expected 80% response rate, we anticipated needing to sample 2 of every 10 days in Matamoros and 2 of every 9 days in Cameron County. Sample days were grouped as 2 consecutive days to minimize interviewer travel time and to allow staggered interviewing schedules by hospital for more efficient hospital coverage. From each of the 10 eligible hospitals (4 in Cameron County, 6 in Matamoros), we systematically selected blocks of 2 consecutive days between August 21 and November 9, 2005. All women who delivered a live infant on these days were sampled. The sample size was expected to allow reasonable assessment of field operations, data collection, and data management activities and the opportunity for collaborative data analysis.

### Data collection instruments

Questionnaire topics were based on USMBHC Healthy Border 2010 (15) objectives related to MCH and chronic disease prevention, including lifestyle and risk behavior, family planning, prenatal health and care, HIV and cervical cancer screening, birth outcomes, child injury, and domestic violence. Questions to obtain demographic information were also included. The questionnaire contained 200 possible data items and was interviewer-administered via laptop computer before the patient's hospital discharge. We reviewed survey instruments from the United States, Mexico, and elsewhere to identify relevant English- and Spanish-language questions, which were translated and modified as needed to reference the pregnancy time period. In each community, we conducted 2 focus groups among currently or recently pregnant adolescents and 2 among adult women to assess respondent ability and willingness to answer questions on the selected topics, familiarity with topic-specific terms, and views on interviews in hospital settings. Results shaped the final bilingual instrument and interviewing method. We formatted the surveillance instrument for electronic data entry using the Census and Survey Processing System (CSPro 2.6, International Programs Center, US Census Bureau, Washington, District of Columbia) and developed paper instruments for back-up purposes. Additional data collection forms developed for data and project management purposes are described in Table 2.

### Training and field operations

Training and field operations were conducted by the United States-Mexico Border Health Association (USMBHA) through a cooperative agreement and with technical assistance from CDC. One field coordinator (FC) and several interviewers (4 in Matamoros and 3 in Cameron County) worked on each side of the border. The Matamoros interviewers and FC spoke Spanish; Cameron County interviewers were bilingual, and the FC spoke English. All interviewers were students or medical professionals and residents of the area. Didactic training for field staff was conducted primarily in Spanish, but all training and reference materials were available in both languages and emphasized general interviewing techniques, sample identification, use of data collection forms, computer use, data entry, editing and processing, data management, and additional supervisory and managerial tasks for FCs. At completion of the 5-day training, skills were assessed and practice interviews were scheduled as needed in hospitals the following week. Interviewers and FCs were compensated for their time in training. During data collection, FCs were employed for 4 months full-time, and interviewers were paid per completed interview.

Interviewers visited each hospital for 3 consecutive days (ie, the 2 sample days plus a third day to complete any outstanding interviews) during each reporting period (ie, the recurring cycle of sampled and nonsampled days for each hospital). On each sample day, interviewers consulted the hospital delivery log book to identify women who had delivered a live infant during the previous 24 hours. As needed, field staff reviewed medical records and communicated with hospital staff to ensure that the sample contained all eligible women. Interviewers recorded information about women included in the sample on a delivery log review form (DLRF), using a unique sample identification number designed to protect the identity of the women. A contact sheet was then prepared for each potential respondent and used to track contact attempts and completed interviews. Interviewers wore white lab coats and a photo badge that identified them as interviewers from the USMBHA. Respondents who were ill or whose babies were severely ill or had died were deferred. Interviews were conducted in Spanish in Matamoros and in the respondent's language of choice in Cameron County. Most interviews occurred in the mother's hospital room, but hallways and other locations were used in instances of hospital overcrowding. Small gifts of appreciation were given to each respondent on completion of the interview.

### Data management and processing

**Figure 3:**
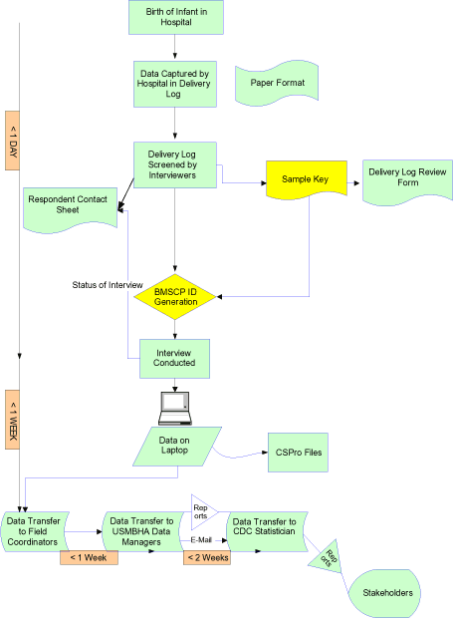
Data Flow in the Brownsville-Matamoros Sister City Project for Women's Health, August 21-November 9, 2005.

Interviewers entered questionnaire data into CSPro files on laptop computers, recorded tracking and respondent contact information on paper forms, and made back-up copies on diskettes (Figure 3). Diskettes and paper tracking forms were given weekly to FCs. FCs reviewed questionnaire data, keyed tracking information into electronic files, observed interviews, and provided feedback to interviewers. They checked hospital delivery log books against DLRFs to assess the completeness of the sample and monitored individual interview response rates and response rates of hospitals. Results of FC assessments and edited interview records were copied to diskette and forwarded to the data manager at the USMBHA. The data manager created cumulative files and performed data quality checks with preprogrammed and ad hoc reports in CSPro. Data were transferred to CDC and stored in 2 places for cross-verification purposes: the personal hard drive of the statistician and the share drive of the Division of Reproductive Health. The personal drive was protected with the user's individual password and the share drive was protected with a network password to maintain data integrity and enable data cleaning and analysis file preparation.

### Procedures to evaluate attributes of the pilot surveillance system

We assessed hospital participation, survey response rate, population coverage, data representativeness, and data quality and incorporated procedures to monitor potential problems in these areas during data collection. To obtain additional information about these and other attributes and feedback from community and government stakeholders about potential usefulness of the data collected, a contracting agency conducted confidential stakeholder interviews during and after the completion of data collection.

**Hospital participation**

To maximize hospital participation and to reduce the burden of data collection on hospital staff, we consulted with hospital administrators and nurses early in the process of protocol development and developed procedures to communicate regularly and to identify potential problems at their onset. We had contingency plans for anticipated events, such as one hospital's transition from a delivery log book to an electronic log system, during the study period. FCs were required to immediately report unanticipated problems to USMBHA.

**Survey response rate**

We computed survey response rates among women sampled in each community and overall. Additional data collected on the respondent contact sheet provided information about the number of contact attempts and reasons for nonresponse.

**Population coverage**

We assessed the degree of noncoverage attributable to 1) the omission of women from the target population who delivered live infants during the study period in hospitals not included in the study and 2) the failure to identify women in the study population who delivered live infants in the study hospitals during the sample days. For comparison and linkage purposes, we accessed Tamaulipas and Texas state records of births that occurred in each community during the study period. As a check of the completeness of birth registration in Matamoros, we merged birth data from the Civil Registry, the vital statistics agency that receives 1 copy of the birth certificate, with those from the Secretariat of Health, which receives another copy.

Potential bias from noncoverage of the target population (no. 1 above) was estimated by comparing distributions of demographic characteristics of all registered live births in Matamoros and Cameron County with births that occurred in study hospitals during the study period and by computing the differences between the proportions for each characteristic. (For these comparisons, Matamoros vital statistics data were provided by the Secretariat of Health and Civil Registry in Tamaulipas, with assistance from the Mexican Institute of Social Security and the Institute for Social Security and Services for State Workers in Tamaulipas; Cameron County data were provided by the Texas Department of State Health Services Pregnancy Risk Assessment Monitoring System program.) To evaluate noncoverage of the study population (no. 2 above), we employed a probabilistic linkage procedure (21) to match the vital records of infants born to women who gave birth in study hospitals on sampling days to BMSCP survey records. For Matamoros records, we used 13 matching variables: folio number; hospital identification number; infant's date of birth, birth weight, and delivery method; and mother's age, marital status, height, weight, education level, number of pregnancies, number of live births, and number of stillbirths. For Cameron County records we used 8 variables: hospital identification number; infant's date of birth, time of birth, birth weight, and delivery method; and mother's age, marital status, and ethnicity (Hispanic). We estimated the study population coverage rate by hospital as follows:

study population coverage rate (%) = (*n*/*n_W_*) x 100 where *n* is the number of women in the BMSCP sample in a specific hospital and *n_W_* is the total number of women in the same hospital who delivered live infants during the sampled days. *n_W _* =* n*_*sv *_+* n*_*os *_+* n*_*ov*_ is the number of women contained in both the BMSCP survey and vital records [*n_SV_*], plus the number of women contained only in the BMSCP survey [*n*_*os*_], plus the number of women contained only in the vital records [*n*_*ov*_].

**Representativeness of pilot data**

A final weight that adjusts for the sampling design, nonresponse rate, noncoverage of the target population, and noncoverage of the study population was computed for each respondent. We assessed data representativeness by comparing the distribution of selected demographic characteristics (age, birth weight, and delivery method) of the BMSCP weighted sample with the distribution of demographic characteristics of the target population using study period birth certificate data from both communities.

**Data quality**

We examined responses to survey questions and any additional information recorded by the interviewer to determine whether questions appeared to have been interpreted correctly by respondents and answered without difficulty. In evaluating each question, we considered the frequency of unknown and missing responses, *other, please specify* responses, adherence to skip patterns, and any comments from respondents, and/or additional input recorded by the interviewer. We flagged questions about outcomes that were rare in this population by noting any response that garnered fewer than 5% of answers to the question. For dichotomous (yes/no) questions, we used a threshold of fewer than 10% of responses.

## Results

### Hospital participation and survey response rate

Each of the 10 hospitals eligible for inclusion agreed to participate in the project and participated throughout the study period. The overall response rate among women sampled was 94.8%. Of total respondents, approximately 92% (484/525) responded to the survey in Cameron County, and approximately 98% (463/474) responded to the survey in Matamoros. Average length of hospital stay varied among hospitals from 6 to 48 hours. Refusal to participate and discharge before the interview were rare (Table 3).

### Population coverage

The study population included 98.3% (2,261/2,301) of all registered live births in Cameron County and 92.7% (2,222/2,398) of all registered live births in Matamoros during the study period (Table 4). Overall differences between the percentage distributions for select demographic characteristics among all registered births and births that occurred in study hospitals were small (≤0.49 percentage points for Cameron County and ≤2.03 percentage points for Matamoros), suggesting that discrepancies between the target and study populations were negligible. Among registered births that reportedly occurred in study hospitals during sample days, 97.4% of mothers in Cameron County and 97.3% of mothers in Matamoros were successfully sampled (data not shown).

### Data representativeness

BMSCP data weighted for sampling design, nonresponse rate, and noncoverage of the target and study populations are compared to vital statistics data  (Table 5). No statistically significant differences in percentage distributions were found for maternal age, birth weight, or delivery method. Differences between unweighted BMSCP data and vital statistics data or weighted data were minimal (data not shown).

### Data quality

The average interview required 35 minutes (29 minutes in English and 37 in Spanish). Few questionnaire items were missing for 5% or more of respondents (Table 6). Respondents had difficulty answering a few questions. For example, 8% of respondents in Cameron County and 19% in Matamoros could not describe their race, and 14% and 9%, respectively, did not know their height. Nearly half of respondents who did not use contraception at first sexual intercourse could not recall the frequency of intercourse before first use. Questions about violence, which were only asked of respondents ≥18 years of age who were alone at the time of interview, were skipped in most interviews (data not shown). Skip patterns throughout the questionnaire appeared to have been followed correctly.

To identify questions that would have limited usefulness in this population, we looked among the dichotomous (yes/no) questions for those in which small numbers of respondents (<10% of total) answered either yes or no. Questions that had such response patterns included ability to obtain needed medical care, injury to the previous child in the past year, smoking during the past 2 years, a previous preterm or low–birth-weight baby, having heard of HIV/AIDS, behavior associated with HIV risk, and among Matamoros women only, not having received prenatal care as early as wanted.

### Timeliness of data collection and processing

Interview and tracking form data were reported to the FC within 1 week of interview. FCs reviewed and transferred data via diskette to USMBHA within 2 weeks of interview, and the USMBHA data manager transferred cumulative data files and associated reports from each reporting period to CDC within 1 month of interview.

### Stability/Reliability

During the study period, power failures, flooding, and a dengue fever outbreak occurred (22). The last event resulted in an acute shortage of hospital rooms for postpartum women. Interviewers conducted the required interviews at the bedside, wherever the bed was located.

### Direct costs of the pilot surveillance system

The total direct costs of conducting 947 interviews were $150,000, $158 per record. Interviewer compensation totaled $30,000, and FC costs were $30,000. The remaining $90,000 supported USMBHA staff, including the data manager, and other expenses. In-kind contributions by local institutions to support field operations and indirect costs to CDC for assistance in implementing the project were not estimated.

## Discussion

Results from this study, as measured by traditional surveillance system evaluation criteria (23,24), indicate that this approach may be effective in similar populations.

### Strengths

Broad-based bilateral participation was important to the success of this pilot program. Hospital participation was 100%. Early hospital concerns regarding demands on staff time, possible patient resistance, and confidentiality were addressed through communication and partnership development. Both US and Mexican local health officials provided in-kind support, such as office space and assistance with accessing local records. Early collaboration with local institutions and the involvement of local project staff were praised by stakeholders in postpilot interviews.

Our response rates were high compared with behavioral risk factor surveys that used other methods. The median response rate in 2001 among US states participating in the Pregnancy Risk Monitoring Assessment Survey (PRAMS), which uses mail and telephone to contact mothers of infants, was 76% (25). Response rates to telephone interviews with adults about health behaviors in the Behavioral Risk Factor Surveillance System in 2005 averaged 51.1% (26), and the response rate to telephone and in-person interviews in the Racial and Ethnic Approaches to Community Health (REACH) communities during 2001-2002 was 53.3% (27). Five states that conducted in-hospital interviews between 1993 and 1996 to boost PRAMS response rates in hard-to-reach, urban populations achieved rates between 71% and 95% (28).

Investigation using birth certificates confirmed that almost all registered births in each community occurred in study hospitals, and almost all registered births that occurred on sample days in study hospitals were captured. Restricting participation to hospitals with at least 100 births per year and using hospital delivery logs to identify women in the sample resulted in exclusion of only a small number of known eligible births and resulted in wide coverage in this population.

Data were highly representative. The weighting factors we calculated were comparatively simple because response and coverage were uniformly good, leaving little chance that any group (eg, adolescents) was significantly underrepresented.

This pilot system used 1 data source and a small number of hospitals. In-hospital interviewing at the time of birth avoided the complications of locating and contacting potential respondents. Computer-assisted personal interviews simplified questionnaire administration and data entry.

Data were rapidly available because respondents were approached immediately after giving birth. This data collection system may become even faster as methods become established and as parallel evaluation steps become unnecessary.

### Weaknesses

The system was stable despite potential disruptions but operated for only 12 weeks. Such a short period of operation is not enough time to draw firm conclusions about system stability. Evaluations of the technical characteristics from an informatics perspective have been conducted and are available from the authors on request.

Lack of privacy during interviews meant that the most sensitive topic, domestic violence, had to be avoided. Furthermore, lack of privacy might have limited the validity of questions on other sensitive topics, such as sexual behavior and abortion history. This problem may be addressed in the future by use of other technologies (eg, audio computer-assisted self-interview).

Questions about race and ethnicity were not answered by substantial proportions of Matamoros respondents, perhaps because these concepts are not relevant on the Mexican side of the border. Questions on height and weight were unanswered by large numbers of US and Mexican women, suggesting that further qualitative study may be needed to identify better measures to assess certain characteristics of this population.

Costs per interview were above typical costs for PRAMS ($129) (Holly B. Shulman, written communication, November 30, 2007), which is conducted primarily by mail. However, the BMSCP study population is harder to reach than the typical PRAMS population, and the response rate was higher. Moreover, substantial state contributions are not included in the PRAMS estimate. Direct costs for in-person REACH interviews in 2005, including interviews in Cameron County, were $350 per interview (Youlian Liao, written communication, November 7, 2007). Continuing federal support for surveys like the BMSCP is not likely, but local health agencies may find ways to share or reduce the direct costs (eg, by having student nurses conduct interviews).

### Areas not evaluated

Some characteristics of the surveillance system could not be assessed. The data collection instrument did not change during the brief period of the pilot, so flexibility could not be demonstrated. No provision was made to test the validity of the data in this setting, although many of the questions have been tested and validated in other surveys. Only basic data quality characteristics were evaluated. The opportunity to use the system in other border communities has not occurred, so its generalizability remains untested.

Measuring the utility of the data that was collected is premature. Pilot data proved to be of high enough quality to justify analysis. Public health agencies in Texas and Tamaulipas are collaborating on initial analyses (29-33). Texas Department of State Health Services staff are preparing the data file for public use, and USMBHA, with assistance from collaborating health institutions, will maintain the data and oversee their release for additional analysis. Documenting further analyses, disseminating results, and assessing the effect of the data on programs and policies in the region will be important.

### Conclusions

Implementation of the BMSCP method depends on the availability of sufficient resources. The system may have to adjust to lean funding by being employed in only a rotating sample of communities or by being conducted at multi-year intervals. Oversampling of some segments of the population (eg, adolescent mothers) or some adverse outcomes (eg, preterm birth) should be considered. The importance of conducting at least minimal surveillance for reproductive health behaviors is likely to grow with the growing border population. Moreover, community characteristics, such as limited access to telephones and cross-border mobility, are unlikely to change, continuing to limit the effectiveness of more traditional surveillance methods in this region.

## Figures and Tables

**Table 1:** Institutional Collaborators and Primary Areas of Activity, Brownsville-Matamoros Sister City Project for Women's Health, Cameron County, Texas, and Matamoros, Tamaulipas, Mexico, 2003-2006

Collaboration	Activity^a^			

	Protocol Development	Field Staff Training	Data Collection	Evaluation Procedures

**Government Collaborators**				
**Mexico**				
Secretary of Health, Tamaulipas	X	–	X	X
Government Workers' Social Security and Services Institute, Tamaulipas	X	–	X	X
Mexican Institute for Social Security, Tamaulipas	X	–	X	X
Tamaulipas Civil Registry	–	–	–	X
National Institute of Statistics and Geographic Information, Tamaulipas	–	–	–	X
Secretary of Health, Mexico: National Center for Epidemiology Control and Disease Prevention; National Center for Health Promotion; National Center for Gender Equity and Reproductive Health	X	–	–	X
**United States**				
Texas Department of State Health Services	X	–	X	X
Centers for Disease Control and Prevention: National Center for Chronic Disease Prevention and Health Promotion; National Center for Health Statistics	X	X	X	X
Cameron County Health Department	X	–	–	–
City of Brownsville Department of Public Health	X	–	–	–
**Binational**				
United States-Mexico Border Health Commission	X	–	–	–

**Community and Academic Collaborators**				

**Mexico**				
Hospital General de Matamoros	X	–	X	–
Instituto Mexicano del Seguro Social Hospital General No. 13	X	–	X	–
Matamoros Hospital Clínica, Dr. Manuel F. Rodríguez Brayda	X	–	X	–
Hospital Guadalupe	X	–	X	–
Centro de Orientación Familiar de Matamoros	X	–	X	–
Centro de Especialidades Médico Quirúrgicas	X	–	X	–
**United States**				
University of Texas School of Public Health, Brownsville Regional Campus	X	–	–	–
University of Texas and Texas Southmost College, Brownsville	X	–	X	–
Valley Baptist Medical Center, Harlingen	X	–	X	–
Valley Baptist Medical Center, Brownsville	X	–	X	–
Valley Regional Medical Center	X	–	X	–
Harlingen Medical Center	X	–	X	–
Cameron Park Cultural Center	X	–	–	–
Brownsville Community Health Center	X	–	–	–
**Binational**				
United States Border Health Association	X	X	X	–

a An "X" indicates that the collaborator took part in the activity; a dash indicates that the collaborator did not take part in the activity.

**Table 2:** Descriptions of Interviewer and Field Coordinator Forms, Cameron County, Texas, and Matamoros, Tamaulipas, Mexico, Brownsville-Matamoros Sister City Project for Women's Health, August 21-November 9, 2005

**Form Type**	Purpose of Form
Delivery log review form	To record identified sampled births and interview status; served as link to sample key.
Sample key	To record identified sample and unique sample number (BMSCP ID); stamped "Confidential."
Respondent contact sheet	To record contact attempts and outcomes.
Interviewer feedback form	To gather feedback from interviewers at project's completion on their experience with and assessment of various aspects of operations.
Training assessment form	To gather feedback from interviewers and field coordinators on training strengths and weaknesses.
Technical assistance form	To request technical assistance from USMBHA and provide details about type of assistance needed.
Weekly hospital report form	To provide weekly summaries to USMBHA of hospital-specific observations, issues, problems, recommendations, and actions taken.
Interviewer observation form	To record details during observations of interviewers on strengths and weaknesses of interviewer performance and to identify areas for review or retraining.
Questionnaire error form	To describe errors found during questionnaire data entry or editing and any corrective actions taken.

Abbreviations: BMSCP, Brownsville-Matamoros Sister City Project for Women's Health; USMBHA, United States-Mexico Border Health Association.

**Table 3:** Interview Response Rates, Brownsville-Matamoros Sister City Project for Women's Health, Cameron County, Texas, and Matamoros, Tamaulipas, Mexico, August 21-November 9, 2005

**Respondent Characteristic**	No. of Respondents in Cameron County (%)	No. of Respondents in Matamoros (%)	Total No. of Respondents (%)
Completed interview	484 (92.2)	463 (97.7)	947 (94.8)
Refused interview	10 (1.9)	0	10 (1.0)
Deferred/not located	31 (5.9)	11 (2.3)	42 (4.2)
Sample total	525 (100.0)	474 (100.0)	999 (100.0)

**Table 4:** Selected Characteristics Among All Registered Births and Registered Births That Occurred in Study Hospitals, Brownsville-Matamoros Sister City Project for Women's Health, Cameron County, Texas, and Matamoros, Tamaulipas, Mexico, August 21-November 9, 2005

Characteristic	Cameron County			Matamoros		

	All Registered Live Births (N = 2,301)^a^, %	Registered Live Births in Study Hospitals (N = 2,261)^a^, %	Difference^b^	All Registered Live Births (N = 2,398)^a^, %	Registered Live Births in Study Hospitals (N = 2,222)^a^, %	Difference^b^
**Age of mother, y**						
<20	16.8	17.0	−0.20	19.0	19.6	−0.56
20-24	27.7	27.9	−0.18	29.4	29.8	−0.34
25-29	27.2	27.0	0.14	27.1	27.0	0.15
≥30	28.3	28.1	0.26	24.4	23.7	0.75
**Infant sex**						
Female	49.9	49.8	0.09	49.6	50.4	−0.89
Male	50.1	50.2	−0.09	50.4	49.6	0.89
**Birth weight, g**						
<2,500	7.5	7.6	−0.04	6.0	6.1	−0.07
2,500-2,999	21.9	21.8	0.01	20.0	20.2	−0.23
3,000-3,499	44.4	44.5	−0.12	40.9	40.8	0.06
3,500-3,999	21.3	21.3	0.02	26.7	26.5	0.20
≥4,000	4.9	4.8	0.13	6.5	6.4	0.03
**Delivery method**						
Cesarean	44.9	44.8	0.14	46.5	44.5	2.03
Vaginal	55.1	55.2	−0.14	53.5	55.5	−2.03
**Mother's marital status, Matamoros**						
Married	NA	NA	NA	53.2	52.0	1.23
Single	NA	NA	NA	9.07	9.39	−0.32
Other	NA	NA	NA	37.69	38.60	−0.91
**Mother's marital status, Cameron County**						
Not married	60.2	59.8	0.40	NA	NA	NA
Married	39.8	40.2	−0.40	NA	NA	NA
**Mother's no. of pregnancies, Matamoros**						
1	NA	NA	NA	33.3	33.3	0.06
2	NA	NA	NA	28.7	28.4	0.29
3	NA	NA	NA	21.2	20.9	0.30
≥4	NA	NA	NA	16.8	17.4	−0.66
**Mother's no. of previous births, Cameron County**						
0	32.1	32.2	−0.08	NA	NA	NA
1	30.2	29.9	0.26	NA	NA	NA
2	22.3	22.3	−0.05	NA	NA	NA
≥3	15.4	15.5	−0.14	NA	NA	NA
**Maternal education level, Matamoros**						
Primary or less	NA	NA	NA	29.1	30.0	−0.87
Secondary	NA	NA	NA	41.7	42.9	−1.16
Preparatory	NA	NA	NA	20.1	19.7	0.41
Professional	NA	NA	NA	9.1	7.5	1.63
**Maternal education, y, Cameron County**						
0-8	13.7	13.7	−0.03	NA	NA	NA
9-11	31.4	31.5	−0.07	NA	NA	NA
12	30.4	30.9	−0.49	NA	NA	NA
13-15	18.0	17.7	0.38	NA	NA	NA
≥16	6.4	6.3	0.19	NA	NA	NA

Abbreviations: NA, not applicable.

a Because of missing data for some characteristics, birth certificate record counts vary across characteristics.

b Difference = Percentage of all registered births minus percentage of registered births that occurred at study hospitals.

**Table 5:** Distribution of Weighted Percentages of Selected Characteristics Among All Registered Births and Births to Survey Participants^a^, Brownsville-Matamoros Sister City Project for Women's Health, Cameron County, Texas, and Matamoros, Tamaulipas, Mexico, August 21-November 9, 2005

Characteristic	Cameron County		Matamoros	

	% of All Registered Births^b^	BMSCP Survey Births, Weighted% (95% CI)	% of All Registered Births^c^	BMSCP Survey Births, Weighted% (95% CI)
**Age of mother, y**				
<20	16.8	14.9 (12.0-17.9)	19.0	19.2 (16.3-22.2)
20-24	27.7	30.2 (26.7-33.8)	29.5	32.2 (28.0-36.4)
25-29	27.2	26.3 (23.0-29.6)	27.1	27.7 (24.6-30.7)
≥30	28.3	28.6 (24.4-32.8)	24.4	20.9 (16.8-25.1)
**Birth weight, g**				
<2,500	7.5	8.5 (5.8-11.2)	6.0	5.0 (3.5-6.6)
2,500-2,999	21.9	23.5 (19.9-27.2)	20.0	20.7 (17.8-23.6)
3,000-3,499	44.4	43.1 (38.4-47.8)	40.9	42.0 (38.6-45.4)
3,500-3,999	21.3	20.7 (17.5-23.9)	26.7	25.0 (21.5-28.5)
≥4,000	4.9	4.2 (2.5-5.9)	6.5	7.4 (5.3-9.4)
**Delivery method**				
Cesarean	44.9	43.5 (39.7-47.3)	46.5	44.3 (41.0-47.6)
Vaginal	55.1	56.5 (52.7-60.3)	53.5	55.7 (52.4-59.0)

Abbreviations: BMSCP, Brownsville-Matamoros Sister City Project for Women's Health.

a BMSCP data weighted for sampling design, nonresponse rate, and noncoverage of the target and study populations are shown in comparison to vital statistics data.

b A total of 2,301 births were registered in Cameron County during the study period.

c A total of 2,398 births were registered in Matamoros during the study period.

**Table 6:** Item Nonresponse Rates Among Questionnaire Items With Missing Data for 5% or More Respondents, Brownsville-Matamoros Sister City Project for Women's Health, Cameron County, Texas, and Matamoros, Tamaulipas, Mexico, August 21-November 9, 2005^a^

Questionnaire Items	Cameron County		Matamoros	

	% of Participants Who Responded "Unknown"	% of Participants Who Refused to Answer	% of Participants Who Responded "Unknown"	% of Participants Who Refused to Answer
Number of times had sex before first birth control use	–	–	39	0
Age at first birth control use	9	1	–	–
Had an HIV test during pregnancy	–	–	6	0
Physical activity/time spent walking	7	0	–	0
Height	14	0	9	0
Prepregnancy weight	6	0	2	13
Race	8	0	19	0
Hispanic/Latina origin	–	–	14	0

a A dash indicates that less than 5% of records had missing responses for this questionnaire item.

## References

[1] Peach J, Williams J (2003). Population dynamics of the US-Mexican border region.

[2] Romero F (2008). Hyper-border: the contemporary United States-Mexico border and its future.

[3] Torrans T (2000). Forging the tortilla curtain: cultural drift and change along the United States-Mexico border from the Spanish era to the present.

[4] Anderson JB, Gerber J (2007). Fifty years of change on the United States-Mexico border: growth, development, and quality of life.

[5] (2006). Centers for Disease Control and Prevention. Quickstats. Percentage of adults aged >18 years without health insurance coverage by ethnicity — United States and counties along the United States-Mexico Border, 2000-2003. MMWR.

[6] Current population survey. Annual social and economic supplement. Table hi05. Health insurance coverage status and type of coverage by state and age for all people: 2006.

[7] (2006). At the crossroads: US/Mexico border counties in transition.

[8] Centers for Disease Control and Prevention. VitalStats. (Custom data request).

[9] Centers for Disease Control and Prevention. Atlas of reproductive health.

[10] (2002). México: frontera norte saludable.

[11] Programa sectorial de salud 2007-2012.

[12] (2007). Centers for Disease Control and Prevention. Sexually transmitted disease surveillance, 2006.

[13] Rangel MG, Martínez-Donate AP, Hovell MF, Santibáñez J, Sipan CL, Izazola-Licea JA (2006). Prevalence of risk factors for HIV infection among Mexican migrants and immigrants: probability survey in the North border of Mexico. Salud Publica Mex.

[14] Strathdee SA, Lozada R, Semple SJ, Orozovich P, Pu M, Staines-Orozco H (2008). Characteristics of female sex workers with US clients in two Mexico-US border cities. Sex Transm Dis.

[15] (2003). Healthy border 2010: an agenda for improving health on the United States-Mexico Border.

[16] Warner DC, Jahnke LR (2003). US/Mexico health issues: the Texas Rio Grande Valley.

[17] Shuler J United States-Mexico border philanthropy partnership and the digital divide.

[18] (2005). Housing in the colonias.

[19] The County Information Project, Texas Association of Counties. Cameron County profile.

[20] II conteo de población y vivienda 2005: resultados definitivos, tabulados básicos. Tabla población 2: población total por municipio, edad desplegada y grupos quinquenales de edad según sexo Aguascalientes (MX) Instituto Nacional de Estadística Geografía e Informática Accessed February 4, 2008 http://www.inegi.gob.mx/est/contenidos/espanol/sistemas/conteo2005/datos/28/excel/cpv28_pob_2.xls .

[21] Smalls M, Kendrick S, Armitage P, Colton T (1998). Record linkage. Encyclopedia of biostatistics.

[22] Centers for Disease Control and Prevention (2007). Dengue hemorrhagic fever — U.S.-Mexico border, 2005. MMWR Morb Mortal Wkly Rep.

[23] Centers for Disease Control and Prevention (2001). Updated guidelines for evaluating public health surveillance systems: recommendations from the Guidelines Working Group. MMWR Recomm Rep.

[24] Romaguera RA, German RR, Klaucke DN, Teutsch SM, Churchill RE (2000). Evaluating public health surveillance. Principles and practice of public health surveillance.

[25] Shulman HB, Gilbert BC, Msphbrenda CG, Lansky A (2006). The Pregnancy Risk Assessment Monitoring System (PRAMS): current methods and evaluation of 2001 response rates. Public Health Rep.

[26] 2005 BRFSS summary data quality report.

[27] Liao Y, Tucker P, Okoro CA, Giles WH, Mokdad AH, Harris VB (2004). REACH 2010 surveillance for health status in minority communities — United States, 2001-2002. MMWR Surveill Summ.

[28] Shulman HB, Johnson C Hospital-based supplementation as a means of improving response in a hard-to-reach population. Proceeding of the International Conference on Survey Nonresponse.

[29] Castrucci BC, Piña Carrizales LE, D'Angelo DV, McDonald JA, Foulkes H, Ahluwalia IB (2008). Attempted Breastfeeding Before Hospital Discharge on Both Sides of the US-Mexico Border, 2005: The Brownsville-Matamoros Sister City Project for Women’s Health. Prev Chronic Dis.

[30] Galván González, Mirchandani GG, McDonald JA, Ruiz M, Echegollen Guzmán, Castrucci BC (2008). Characteristics of Young Women Who Gave Birth in the US-Mexico Border Region, 2005: The Brownsville-Matamoros Sister City Project for Women’s Health. Prev Chronic Dis.

[31] Gossman GL, Carrillo Garza CA, Johnson CH, Nichols JJ, Castrucci BC, McDonald JA (2008). Prenatal HIV testing in the US-Mexico border region, 2005: the Brownsville-Matamoros Sister City Project for Women's Health. Prev Chronic Dis.

[32] Robles JL, Lewis KL, Folger SG, Ruiz M, Gossman GL, McDonald JA (2008). Prior Contraceptive Use Among Women Who Gave Birth in the US-Mexico Border Region, 2005: The Brownsville-Matamoros Sister City Project for Women’s Health. Prev Chronic Dis.

[33] Castrucci BC, Echegollen Guzmán A, Saraiya M, Smith BR, Lewis KL, Coughlin SS (Dis 2008). Cervical cancer screening among women who gave birth in the US-Mexico border region, 2005: the Brownsville-Matamoros Sister City Project for Women's Health. Prev Chronic Dis.

